# Editorial: Insights in coronavirus disease (COVID-19) - surveillance, prevention and treatment

**DOI:** 10.3389/fpubh.2022.998998

**Published:** 2022-09-29

**Authors:** Zisis Kozlakidis, Marc J. Struelens

**Affiliations:** ^1^International Agency for Research on Cancer, World Health Organization, Lyon, France; ^2^Faculty of Medicine, Universite Libre de Bruxelles, Brussels, Belgium

**Keywords:** COVID-19, infectious diseases, surveillance, prevention, vaccine, screening, treatment

## Introduction

The achievements made by scientists in the fast-growing field of COVID-19 have been exceptional over the last 2 years, leading to major advancements in understanding and managing the new disease, but also transforming the fields of infectious diseases and public health in general. Following the SARS-CoV-2 outbreak and subsequent pandemic, the multi-disciplinary work of researchers worldwide has provided an in depth understanding of COVID-19 pathogenesis, of clinical treatments and outcomes, of models and dynamics governing disease spread, period of infectivity and containment interventions. Immunological research focused on the understanding of acquired immunity, and development of innovative vaccines and effective vaccination schemes, which have greatly improved disease outcomes across the globe. The required rapid processing and dissemination of scientific information would not have been possible if not for the special focus and attention given by scientific journals such as Frontiers in Public Health and Frontiers in Medicine.

Following on from the success of the first Frontiers COVID-19 Research Topic, featuring 400 original research articles (1), and Volume II of the same title featuring 163 original articles (2), a dedicated COVID-19 Research Topic was opened for submissions, entitled “Insights in Coronavirus Disease (COVID-19) - Surveillance, Prevention and Treatment”. Relative to Volumes I and II, which were broad in scope, this topic, running from June 2021 to January 2022, focused primarily on novel developments, current challenges, latest discoveries, and future perspectives in the field of COVID-19. The aim for this article collection was to inspire, inform and provide direction to researchers in the field.

To provide a backdrop, at the time of the launch of this Research Topic in June 2021, the first wave of the rollout of population-wide SARS-CoV-2 vaccinations with two doses was nearing completion in most European countries, while a severe lack of vaccine availability was still recorded for resource-restricted settings. Following the vaccination successes in Europe and North America a gradual relaxation of public health restrictions was afforded while many countries worldwide were preparing for resurgences. In total, 176 manuscripts were submitted, 83 (47%) of which were accepted. As of August 2022, the special topic achieved approximately 255,000 views and 31,000 article downloads, with readership distributed across the globe. Frontiers made a significant contribution to the timely generation and distribution of peer-reviewed COVID-19 publications as the publisher of this special topic, as well as many other related topics. At the time of writing, the top five most viewed Research Topics on COVID-19 published in Frontiers in Medicine and Frontiers in Public Health (1–5) have generated over 19 million of views, as well as over 8,000 article citations.

Among the five broad areas covered by this Research Topic, primary focus of the accepted manuscripts was on Public Health Response (26), followed by Clinical Management (19), Epidemiology (14). Pathophysiology (12) and Screening Methods (12). The accepted submissions comprised of Original Research (50), Brief Research Report (11), Reviews (8), Opinion (5), Case Report (4), Methods (2), Community Case Study (1), Hypothesis and Theory (1) and Perspective (1).

## Public health response and vaccines

The understanding and comparison of public health responses has been vital in the design and quick adaptation of effective mitigation strategies. A number of manuscripts shared details of investigations of SARS-CoV-2 transmission patterns and non-pharmaceutical control interventions implemented in defined geographies, such as China (Lu et al.), the Czech Republic (Dziedzinska et al.), Germany (Kapnsner et al.), Portugal (Leite et al.), Romania (Dascalu et al.), Saudi Arabia (Hakeem et al.) and the USA (Bonacci et al.). Awareness about the virus transmission within public education settings and protective practices were also examined in the studies by Lordan et al., Middleton et al., Sombetzki et al., and Qin et al., as well as within areas of precarious housing conditions by Zimmermann et al..

As population-wide vaccination programs were implemented across the world, a number of studies emerged considering the overall COVID-19 vaccination acceptance, providing survey-based quantitative estimates (Al-Qerem and Jarab; Lyu et al.; Zhang et al.). Some of these early data was summarized by the systematic review of Norhayati et al. The perspectives and responses of different communities toward COVID-19 were considered, such as pharmacists being both vaccinated and vaccinators (Turcu-Stiolica et al.); of patient groups being vaccinated (Ahmadi et al.); as well of wider communities within specific geographical areas (Kuo et al.; Liu et al.). Related to the safety of vaccination programs and providing a holistic view of the subject within this special topic, were also the studies describing vaccine reactions in the USA, EU (Montano) and China (Zhu et al.). Finally, the protective properties of breast milk for infants from COVID-19 were outlined in the Hypothesis and Theory study by Quitadamo et al..

As the impact of the COVID-19 pandemic is multi-faceted, some studies presented practical and directly implementable solutions for the many challenges it raised. Drobniewski and Keshavjee analyzed the similarities between COVID-19 and tuberculosis, and how the extensive lessons learned from the older pathogen might transfer to the response against the newer one. Sehgal and Milton discussed how frameworks from occupational health might be applicable in navigating the next, more episodic, phases of the pandemic, while Shi et al. presented the construction and implementation of an intelligent voice-response system for COVID-19 information management.

## Clinical management

The topic of COVID-19 was approached from a clinical management perspective as well, with Priori et al., providing a University Hospital multidisciplinary account from Milan, Italy, of re-organizing inpatient care during the first wave of the pandemic. Some studies on disease progression were in the context of other comorbidities, for example gastro-intestinal symptoms (Chen et al.) and pancreatitis (Fiore et al.). Various approaches to assessing prognostic factors and indicators were reported. Enocsson et al. looked at soluble urokinase Plasminogen Activator Receptor (suPAR) as a specific prognostic biomediator. On the other hand, Ranard et al., looked at a wider set of indicators reporting different patient endotypes that might be prognostic for COVID-19. One study by Kurban et al., provided examples of simple triage tools that can be used to predict severe disease.

Among the articles with a primary focus of clinical management, another main area of interest was the evaluation of various new or concomitant therapies with respect to benefits for COVID-19 progression and outcome. Treatments evaluated included the addition of baricitinib to standard care (Masiá et al.); the effect of tocilizumab monotherapy on biomediators (Hashimoto et al.); the infusion of umbilical cord mesenchymal stromal cells (da Silva et al.); the use of A2 adrenergic receptor agonist (Li et al.; Hamilton et al.); as well as the case report of blood purification for 5 severely ill patients (Chen et al.). Regarding the prevention of symptoms or at least the reduction of symptom severity, studies looked into the supplementation with vitamin D (Arroyo-Díaz et al.) and zinc (Hardigan and Gordon). The breadth of topics covered demonstrate the multitude of approaches still being considered in the effort to improve the current standard of care for COVID-19, as well as indicating the many clinical specialties involved in those investigations. This aspect of plurality in COVID-19 treatments was captured well in the meta-analysis of randomized control trials by Zhang et al., while Wüstner et al., looked into the clinical evidence informing treatment guidelines, specifically on repurposed drugs for hospitalized patients during the early waves of the COVID-19 pandemic, including for corticosteroids, anticoagulants and other.

Accurate, timely COVID-19 diagnosis and tools used for diagnosis was another research area of interest in clinical management, though of reduced frequency as compared with the earlier special topics on COVID-19. Namely, Avgoulea et al. in their brief research report focused on the implementation of rapid point of care diagnostics in a hospital emergency unit. Finally, it has been well-established that the impact of the pandemic has not been limited to physical health and acute disease. Two studies looked at the post COVID-19 sequelae, in general (Makrydakis et al.) and specifically, as they relate to chronic fatigue syndrome (Hohberger et al.). Frontiers journals have initiated distinct special topics on COVID-19 sequelae, especially on emotional/mental health and post COVID syndrome assessment (3).

## Epidemiology

Of the articles primarily focused on Epidemiology, disease surveillance remained an area of focus in order to elucidate the geographical, demographic and behavioral distribution of confirmed cases at specific time points (or over time). As such, there were studies regarding COVID-19 epidemiologic patterns in Burkina Faso (Kaboré et al.), the Ecuadorean army (Ortiz-Prado et al.), the Colombian army (Duque et al.), South Korea (Hong et al.), China (Li et al.), as well as the incidence in cannabis users (Huang et al.). In order to complement the picture on symptomatic case identification, Syangtan et al. focused their systematic review on investigating asymptomatic cases. Finally, the study by Yadav et al. described an outbreak of Nipah virus concurrent to COVID-19 in India, reminding that COVID-19 is cumulative to existing pathogen pressures in many countries.

Accurate disease surveillance remains a cornerstone for healthcare systems for the timely implementation of public health measures as warranted, and for resource and treatment planning relative to available service capacity. Thus, a clear area of interest remained the modeling and testing of prediction models that might be implemented in subsequent waves of the pandemic. These included work by Inglis et al. in estimating the local COVID-19 risk during the pandemic curve decline; a seasonality-aware model by Alser et al.; as well as a model for short-term COVID-19 prediction, based on USA data by Majeed et al. Furthermore, the predictive models by Pei Y et al., and Zuo et al., included vaccination as a co-factor in their framework. Analyzing available data from Germany and Italy and their states and regions (January–June 2020), Morfeld et al. provided an estimate of the excess mortality attributable to acute COVID-19 and highlighted limitations of routine demographic surveillance systems. The goal of these investigations was to achieve a better understanding of the individuals/groups with increased risk, and thus, prioritizing any public health interventions to decrease the transmission risk to susceptible populations.

## Screening methods

Asymptomatic and pre-symptomatic transmission of SARS-CoV-2 has introduced a greater degree of difficulty and uncertainty for monitoring the spread of the infection into new clusters. Thus, population-wide or high-risk group screening policies have been implemented at various scales in many countries. The studies included in this special topic describe the implementation of such screening policies at national borders (Chua et al.), at schools (Simas et al.), at healthcare facilities (Raimann et al.). As with any diagnostics, a greater than acceptable threshold of false-positive or false-negative outcome, can cause considerable damage both at individual and public health levels. Therefore, a number of studies focused on the evaluation of the individual performance of such test and/or methodologies (Alghounaim et al.; Yingtaweesittikul et al.; Alqahtani et al.; Eskobar et al.; Wertenauer et al.; Fernández-Rivas et al.). The investigation of saliva as a suitable detection fluid was discussed by Pierri et al.; while the implementation of ELISA-based seroprevalence for the understanding of asymptomatic transmission levels was considered by the studies of Sherman et al., and Breedon et al..

## Pathophysiology

Understanding the pathophysiology of COVID-19 and its many clinical manifestations is crucial in dealing with the severe forms of the disease, identifying individuals with increased risk, and taking timely action toward development and/or implementation of appropriate treatments. Hayes et al. provided a scoping review of over 100 persistent symptoms of long COVID-19; while the study by Cheng et al. described a text clustering method to identify symptom clustering. The systematic review by Sodeifian et al. looked into the drug-induced liver injury in COVID-19 patients; and Zhang et al. investigated liver fibrosis scores and clinical outcomes in COVID-19 patients. There were two Opinion papers in this special topic, one by Kozlakidis, considering the relative lack of evidence of genomic recombination for SARS-CoV-2; and another by Dubina, considering a potential non-immune prophylaxis against COVID-19 by targeting tolerance for Angiotensin II.

The link between vitamin intake and COVID-19 clinical outcomes remains a focal point of research. To this end the systematic review by Teshome et al. investigated the impact of vitamin D on COVID-19 infection; while Chen et al. investigated the relationship between plasma vitamin C and COVID-19 susceptibility and severity. The investigation of such relationships allows the deeper understanding of molecular mechanisms and their link to clinical outcomes. The relationship between platelet count and COVID-19 mortality was investigated by Yang et al.; while another relationship, between cellular and humoral immune responses after two doses of vaccination were studied by Mangia et al. The brief research report by Jimah et al. focused on the monitoring of COVID-19 positive pregnant women. Finally, Holm-Jacobsen et al. investigated the clinical implications of rectal SARS-CoV-2 shedding in Danish patients.

## Conclusions

As the COVID-19 pandemic continues and as the SARS-CoV-2 virus continues to evolve and produce a greater number of variants, it is likely that the case numbers and hospital admissions will persist their fluctuations globally. Thus, it is not surprising that epidemiological studies continue to feature strongly in the published scientific literature as understanding of the changing dynamics of COVID-19 remains a public health priority. To this end, the greater emphasis on public perceptions, attitudes and behaviors is a welcome development, further strengthening public health responses and gradually involving an active participation of “citizen scientists” as advocated previously (6).

Building on understanding the pathophysiology and epidemiology of the disease, the development of efficacious vaccines and treatments have made a clear difference in addressing severe forms of the disease and mitigating its public health impact. More contagious and/or virulent variants of the virus that are able to evade acquired immunity may continuously emerge, and their widespread dissemination is of concern in the near future. Novel and/or improved virus screening methods, therapeutics and vaccines are likely to remain key tools toward pandemic mitigation. The cumulative burden of “long COVID” sequelae as well as psychological/psychiatric impact of the disease and its control measures are crucial topics to study for guiding long term medical and public health management. Therefore, concerted research efforts on current topics remain a crucial part of the continued fight against COVID-19.

## Author contributions

ZK compiled/wrote the first draft. MS contributed to the outline. Both authors reviewed and edited for final revisions. All authors approved the final version for publication.

## Conflict of interest

The authors declare that the research was conducted in the absence of any commercial or financial relationships that could be construed as a potential conflict of interest.

## Publisher's note

All claims expressed in this article are solely those of the authors and do not necessarily represent those of their affiliated organizations, or those of the publisher, the editors and the reviewers. Any product that may be evaluated in this article, or claim that may be made by its manufacturer, is not guaranteed or endorsed by the publisher.
